# *Euphorbia marginata* Alleviate Heavy Metal Ni-Cu Combined Stress by Regulating the Synthesis of Signaling Factors and Flavonoid Organisms

**DOI:** 10.3390/plants14142159

**Published:** 2025-07-13

**Authors:** Xudan Zhou, Tian Jin, Te Li, Yue An, Xintian Dai, Chunli Zhao, Tongbao Qu

**Affiliations:** Jilin Provincial Key Laboratory of Tree and Grass Genetics and Breeding, College of Forestry and Grassland Science, Jilin Agricultural University, Changchun 130118, China; zhouxudan@jlau.edu.cn (X.Z.); jtxkbjly273@163.com (T.J.); 15140122381@163.com (T.L.); yue.an.jlau@outlook.com (Y.A.); 15135720482@139.com (X.D.)

**Keywords:** heavy metal, *Euphorbia marginata*, plant pathogen interactions, phenylpropane

## Abstract

It is of great importance to explore how plants respond to excess accumulation of Cu and Ni in soil, yet the mechanisms by which *Euphorbia marginata*, a common ornamental plant in China, responds to heavy metal stress remain unclear. In this study, *E. marginata* seedlings were subjected to CK, Ni 500 mg/kg, and Cu 900 mg/kg, with Ni-Cu combined stress, and their growth, physiological indexes, heavy metal accumulation, and their corresponding gene expression were evaluated after 45 d. The results showed that the two heavy metals mainly accumulated in plant roots and severely inhibited root growth, while the combined stress promoted the accumulation of heavy metals to a small extent. Either Cu or Ni stresses inhibit photosynthetic pigment synthesis as well as activate antioxidant and osmoregulatory systems, but there are differences in their effects. Combined stress has a synergistic stress effect, severely damaging the cell membrane structure and leading to dysregulation of antioxidant and osmoregulatory systems. The expression of CDPK, CaMCML, MEKK3/6 signaling factors, UFGT, and COMT was severely suppressed under the combined stresses of Cu and Ni compared to the single stress of both. These results provide evidence of a specific defense response to heavy metal stress in *E. marginata*, which could help guide new research efforts and support the development of strategies for phytoremediation using *E. marginata*.

## 1. Introduction

With the development of industrialization, urbanization, and intensive agriculture, soil heavy metal pollutants, such as Cu and Ni, have become a major environmental challenge due to the non-biodegradability of these metals [1,2,3,4]. Heavy metals persist in the soil as ions [5], such as Ni^2+^ and Cu^2+^. In certain regions of China, soils near Ni-Cu mines demonstrate significant contamination, with concentrations of Ni^2+^ and Cu^2+^ surpassing regulatory thresholds by 2.1% and 4.8%, respectively [6,7]. Both Cu and Ni are essential micronutrients for plant growth, while elevated soil concentrations can negatively impact physiological processes and hinder plant growth. It is essential that targeted planting of highly accumulative ornamental species be implemented in mining areas and that systematic screening of their ecological and esthetic traits be conducted to advance ecologically sustainable soil remediation programs.

Under heavy metal stress, plants activate signaling pathways to implement defense strategies [8]. Excess concentrations of Ni can prove toxic and result in damaging alterations in the metabolism and growth of plants [9,10]. Reduced plant growth due to Ni toxicity has been attributed to the significant reduction in photosynthesis, enzyme activity, membrane stability, and mineral nutrition [11]. Unlike Ni, excessive Cu accumulation in plants reduces chlorophyll biosynthesis, suppresses root and shoot elongation, diminishes biomass, and accelerates senescence [12]. These effects collectively disrupt redox homeostasis and impair nutrient assimilation and photosynthetic efficiency, thereby exerting multifaceted phytotoxic effects on plant growth [13]. Excessive accumulation of metals in plants induces the production of excessive amounts of reactive oxygen species [14], which triggers oxidative degradation of cellular macromolecules (e.g., proteins, lipids, nucleic acids). This oxidative damage subsequently compromises membrane integrity, alters protein conformation, and impairs enzyme function [15]. Fundamental signaling cascades involved in heavy metal stress adaptation, which overlap with responses to abiotic stress, include calcium-regulated pathways, phytohormonal networks, and MAPK-mediated phosphorylation cascades. Calcium signaling functions through sensor proteins, such as calmodulin, and effector kinases, like calcium-dependent protein kinases (CDPKs), which interpret spatial and temporal patterns of Ca^2+^ flux [16]. In roots, phenylpropanoid metabolism begins with the deamination of phenylalanine, leading to the synthesis of various phenolic metabolites [17]. Flavonoid synthesis pathway-related substances, including hydroxycinnamic acids, possess both ROS-scavenging and metal-chelating properties, which help alleviate the effects of abiotic stress. Recent studies mainly focused on plant growth, physiological adaptations, and gene expression variations in response to individual heavy metal stress, with little exploration of the dynamics associated with Ni-Cu co-exposure.

Multiple heavy metal contaminants are generally present in the soil at the same time, and they interact with each other to cause more complex combined stresses on plants. Under the complex conditions of different heavy metal combined stresses, these metal interactions may trigger hormonal growth stimulation while disrupting physiological homeostasis [18]. The phytotoxic responses observed from metal mixtures demonstrate non-additive synergies and antagonisms, which significantly complicate predictions of remediation effectiveness [19,20]. Research on Cu-related combined stresses has focused on the following heavy metals, such as Cr, Cd, and Zn, and they have emphasized surface morphological responses to multiple heavy metal stresses without delving into mechanistic interactions [21]. Seed germination and root vigor are promoted by elevated Cu and Cr concentrations, while chlorophyll biosynthesis and superoxide dismutase (SOD) activity are suppressed [22]. Exposure to both Cu and Cd resulted in reduced plant biomass [23]. Whereas, simultaneous exposure to Cu and Zn resulted in increased MDA levels leading to irreversible degradation of membrane lipid bilayers and lipid peroxidation [24], as well as interfering with chlorophyll synthesis [25]. Ni-Cu combined contamination often occurs simultaneously in soils, yet not only is there a paucity of relevant research, but also the molecular mechanisms of how plants respond to heavy metals are not yet clear [26,27,28]. It is thus important to prioritize the identification of species with exceptional tolerance to Ni-Cu and to explore their response mechanisms to heavy metals as an important research goal.

*Euphorbia marginata* Pursh (Euphorbiaceae) is an annual herb native to North America. Its unique leaf color has a high ornamental value, which is why it is widely used in landscaping. Because of its rapid growth and large biomass, it is assumed to have strong resistance to adversity. Nowadays, there are fewer studies on the response to adversity stresses of *E. marginata*. In summary, this study employs growth physiological indices of single Ni or Cu and combined metal stress treatments, with a particular focus on antioxidant and osmoregulatory systems, which were used to elucidate the molecular adaptive strategies of *E. marginata* through comparative transcriptomic analyses. It could help guide new research efforts and support the development of plant restoration strategies. And hypotheses for this research were made as follows: 1. Plant accumulation of heavy metals may be concentrated in the roots or leaves. 2. Excessive Cu and Ni alter the osmotic pressure inside and outside the plant cell, while the entry of excess ions into the cell stimulates the antioxidant system of the plant, resulting in the generation of large quantities of ROS. 3. Under combined stress, the interaction of Cu and Ni may reduce the toxicity of each other, and differential expression of genes different from that of a single stress in the antioxidant and osmoregulatory systems may be observed, leading to the growth of the plant that is superior to that of a single stress.

## 2. Results

### 2.1. Effect of Different Concentrations of Cu^2+^ and Ni^2+^ on Growth Indicators and Heavy Metal Content of E. marginata

**Figure 1:**
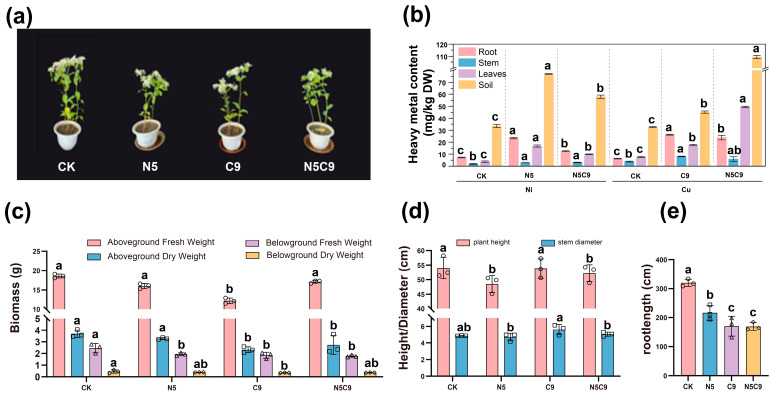
Effects of different concentrations of Cu^2+^ and Ni^2+^ on the growth indexes and heavy metal contents of silver-bordered crocus at various parts of the *E. marginata* under stress. Morphology at different concentrations (**a**), heavy metal content in different parts (**b**), biomass (**c**), plant height and stem diameter (**d**), and specific root length (**e**). Different lowercase letters indicate significant differences between treatments (*p* < 0.05); N5: Ni500; C9: Cu900; N5C9: Ni500+Cu900; DW: dry weight, Different circles, squares and triangles represent the specific values of three biological repetitions within the same group.

Under single Ni or Cu stress, all growth parameters (root length, biomass) decreased significantly versus CK (*p* < 0.05; Figure 1), while stem diameter increased. Heavy metal analyses revealed that Ni accumulated predominantly in roots under Ni stress (23.42 ± 0.60 mg/kg; *p* < 0.05), exceeding both CK and combined stress levels. Under combined stress, Ni contents in roots and leaves remained higher than CK but lower than under Ni stress, indicating altered accumulation of metal in *E. marginata*. Cu accumulation in roots increased in the combined stress group (Ni500+Cu900) compared to the control (CK) group. Ni or Cu contents in soil differed significantly among treatments (*p* < 0.05), though soil Cu remained stable across groups. Enrichment and transfer coefficients, key indices of plant metal accumulation capacity, were elevated under Ni stress versus CK (Table S4). Notably, the transfer coefficient peaked in the combined stress group, suggesting enhanced internal metal translocation as an adaptive response to environmental pressure.

### 2.2. Effect of Different Concentrations of Cu^2+^ and Ni^2+^ on Oxidation, Osmosis, and Photosystems of E. marginata

**Figure 2:**
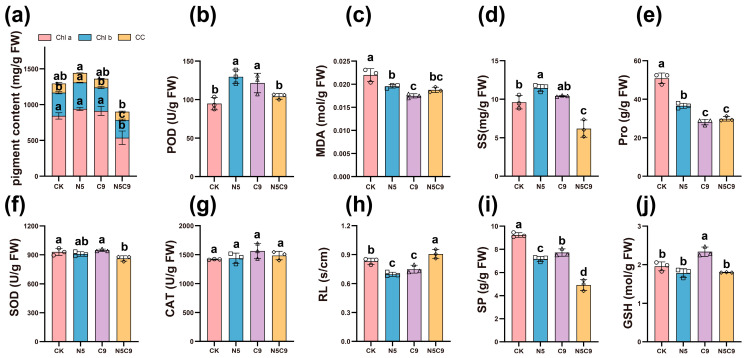
Effect of different concentrations of Ni^2+^ and Cu^2+^ on physiological indices of *E. marginata* under stress. Photosynthetic pigment content: chlorophyll a (Chl a), chlorophyll b (Chl b), carotenoid (CC), (**a**); peroxidase (POD), (**b**); malondialdehyde (MDA), (**c**); soluble sugar (SS), (**d**); proline (Pro), (**e**); superoxide dismutase (SOD), (**f**); catalase (CAT), (**g**); relative electrical conductivity (RL), (**h**); soluble protein (SP), (**i**); and glutathione (GSH), (**j**). Different lower case letters indicate significant differences between treatments (*p* < 0.05); N5: Ni500; C9: Cu900; N5C9: Ni500+Cu900; FW: fresh weight, Different circles, squares and triangles represent the specific values of three biological repetitions within the same group.

Ni-Cu stress damaged the integrity of chloroplasts, and the contents of various types of photosynthetic pigments decreased. The contents of chlorophyll a, chlorophyll b, and carotenoids in different groups were studied and determined. Compared with the CK group, the contents of chlorophyll a, chlorophyll b, and carotenoids in the Ni500 group increased by 11.46%, 13.84%, and 4.78%, respectively (*p* < 0.05), while the contents of chlorophyll a, chlorophyll b, and carotenoids in the Cu900 group increased by 8.18%, 0.97%, and 1.17%, respectively, with no significant difference. However, combined stress significantly reduced chlorophyll a (36.28%), chlorophyll b (23.50%), and carotenoids (9.43%) versus CK (*p* < 0.05) (Figure 2a). Compared with the CK group, the activities of antioxidant enzymes in the Ni500 group (SOD: 36.87%, POD: 0.90%, CAT: 1.42%) and the Cu900 group (SOD: 28.31%, POD: 1.83%, CAT: 10.40%) were increased. Among them, the change in POD activity was significant (*p* < 0.05), but in the Ni500+Cu900 group, the increase was only 10%, which was significantly lower than the single stress of the Ni and Cu. Relative conductivity declined across stress groups, while MDA decreased by 11.08% (Cu) and 20.49% (Ni) versus CK, remaining lower under combined stress (14.57%) (Figure 2b,c,f,g,h).Osmolytes (SS, SP, and Pro) showed treatment-specific variations: SS and SP decreased under combined stress (*p* < 0.05), whereas Pro declined under Ni stress. These shifts suggest osmoregulatory adaptations to divergent stress conditions (Figure 2d,e,i). The GSH content under combined stress (Group N5C9) was significantly decreased (*p* < 0.05) by 8.6% and 29.6%, respectively, compared with that under single stress (Group Ni500 and Group Cu900) (Figure 2j). It is speculated that when excessive ROS is produced and the chelation of PCs continuously consumes GSH, the level of GSH decreases, resulting in weakened antioxidant capacity [29].

### 2.3. Redundancy and Interaction Analyses of Physiological Indicators Affecting Growth Changes Under Different Concentrations of Ni^2+^ and Cu^2+^ Treatments

**Figure 3:**
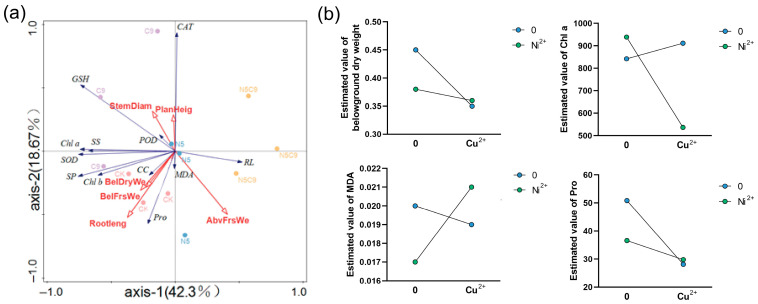
Redundancy and interaction analyses of physiological indicators affecting growth changes under different concentrations of Ni^2+^ and Cu^2+^ treatments. (**a**) redundancy analysis; (**b**) interaction analysis. N5: Ni500; C9: Cu900; N5C9: Ni500+Cu900; Planheig: plant height; Rootleng: root length; AbvFrsWe: aboveground fresh weight; AbvDryWe: aboveground dry weight; BelFrsWe: belowground fresh weight; BelDryWe: belowground dry weight.

The redundancy analysis of physiological indicators affecting growth changes under different treatment conditions is shown in Figure 3a. The eigenvalues for axes I to IV under the treatment changes were Cu900, Ni500+Cu900, Ni500, and CK. The growth indicators, ranked by their contribution, were aboveground fresh weight, root length, and belowground dry weight, with aboveground fresh weight contributing 42.3%. The physiological indicators were ranked by importance as follows: GSH > CAT > Chl a > SS. This factor had the greatest influence on species variation. Based on their effects on species, the treatments were ranked in terms of importance as follows: Cu900 > Ni500+Cu900 > Ni500 > CK.

Interaction analyses of *E. marginata* indicators under Ni-Cu combined stress (Figure 3b, Table S5) revealed significant multiplicative effects on biomass, photosynthetic pigments, MDA, and antioxidant enzymes (*p* < 0.05), demonstrating non-additive synergistic or antagonistic interactions. Belowground dry weight showed significant Ni-Cu main effects (*p* < 0.05), with combined stress intensifying growth inhibition. While Ni alone had non-significant main effects on chlorophyll a (*p* < 0.05), Cu and Ni-Cu interactions significantly reduced it (*p* < 0.05), indicating Cu-driven photosynthetic impairment exacerbated by Ni. MDA responded significantly to both metals individually and synergistically (*p* < 0.05), confirming combined stress amplifies membrane peroxidation. Most antioxidant enzymes (SOD, POD, CAT) exhibited significant interaction effects (*p* < 0.05), reflecting nonlinear oxidative stress regulation under combined exposure.

### 2.4. Transcriptomic Analysis of the Response of E. marginata to Single and Combined Ni-Cu Stresses

#### 2.4.1. Transcriptome Sequencing Data and Quality Evaluation

**Figure 4:**
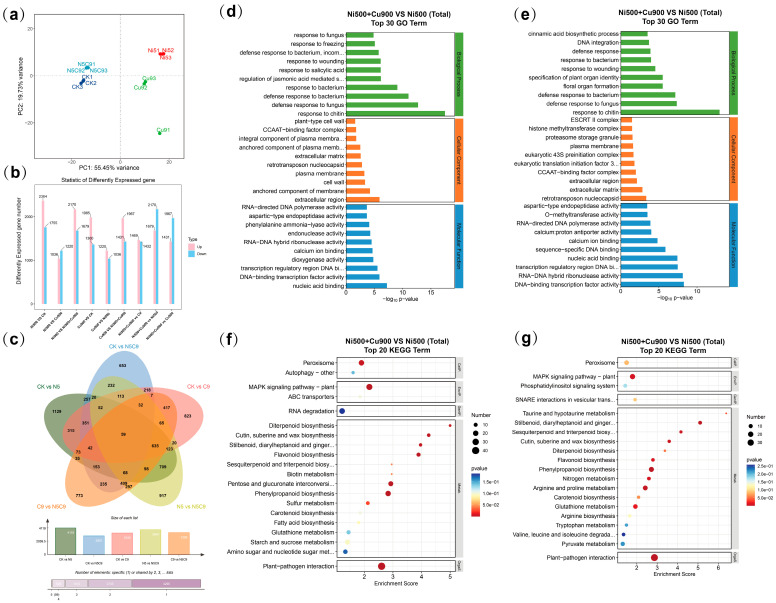
RNA-seq-based effects of Ni-Cu stress on *E. marginata* genomic expression profiles. (**a**) Plot of principal component analysis of transcriptome data of the group of CK, Ni500, Cu900, and Ni500+Cu900. (**b**) Number of DEGs under different groups. Red color indicates gene up-regulation and blue color indicates gene down-regulation. (**c**) Venn diagram of DEGs under different groups. (**d**) Bubble plots of GO enrichment analysis of TOP30 in Ni stress and combined stress. (**e**) Bubble plots of GO enrichment analysis of TOP30 in Cu stress and combined stress. (**f**) Bubble plots of KEGG enrichment analysis of TOP20 in Ni stress and combined stress. (**g**) Bubble plots of KEGG enrichment analysis of TOP20 in Cu stress and combined stress; larger bubbles indicate higher DEG enrichment.

A total of 35.70 GB of raw data were obtained from high-throughput sequencing of the four groups (CK, Ni500, Cu900, and Ni500+Cu900). The raw reads were filtered to generate clean reads, ensuring the quality of transcriptome data analysis. We used the Pearson correlation coefficient (r) as the evaluation index for the correlation between samples. The correlation heatmap between the samples is presented (Figure S1). The results indicate that the samples have good reproducibility, and the reliability of the differentially expressed genes meets the required standards. Principal component analysis (PCA) evaluated the dispersion of the samples, as illustrated in Figure 4a. Additionally, the GC content of each sample was greater than 44%, with Q20 values exceeding 97% and Q30 values exceeding 93%. These results indicate that the sequencing quality is good and suitable for subsequent reference genome comparison analysis.

#### 2.4.2. Quantitative Analysis of Differentially Expressed Genes

*E. marginata* were screened for differential genes in each treatment group (CK, Ni500, Cu900, Ni500+Cu900) under heavy metal stress according to the conditions of |Log_2_Fold Change| > 1; *p*-adjust < 0.05 (Figure 4b). The results showed that in the Ni500+Cu900 and Ni500 groups, a total of 3849 differential genes were screened, of which 2170 were up-regulated and 1679 were down-regulated. In the comparison between the Ni500+Cu900 and Cu900 groups (Figure S2), a total of 3398 differential genes were screened, of which 1431 were up-regulated and 1967 were down-regulated. 3398 differential genes were screened, of which 1431 were up-regulated and 1967 were down-regulated (Figure S3).

#### 2.4.3. GO and KEGG Enrichment Analysis

The GO enrichment analysis indicated that the set of DEGs in the Ni500+Cu900 vs. Ni500 comparison was significantly enriched in 187 pathways (*p* < 0.05), including 107 biological processes, 13 cellular components, and 67 molecular functions. The enriched gene ontology terms included ‘cell wall macromolecule catabolic process’ (GO:0016998), ‘glucosinolate transport’ (GO:1901349), ‘aspartic-type endopeptidase activity’ (GO:0004190), ‘glutathione transferase activity’ (GO:0004364), ‘peroxidase activity’ (GO:0004601), and ‘chloroplast thylakoid membrane protein complex’ (GO:0098807). The set of DEGs in the Ni500+Cu900 vs. Cu900 comparison was significantly enriched in 191 pathways (*p* < 0.05), including 108 biological processes, 16 cellular components, and 67 molecular functions. The significantly enriched gene ontology terms for the Ni500+Cu900 vs. Cu900 comparison included ‘cell wall macromolecule catabolic process’ (GO:0016998), ‘chlorophyll catabolic process’ (GO:0015996), ‘chloroplast thylakoid membrane protein complex’ (GO:0098807), and ‘cinnamyl-alcohol dehydrogenase activity’ (GO:0045551).

The KEGG enrichment analysis indicated that these pathways were enriched in seven pathways under Ni500 vs. Ni500+Cu900 and Cu900 vs. Ni500+Cu900 including ‘Plant–pathogen interaction’ (ko04626), ‘Phenylpropanoid biosynthesis’ (ko00940), ‘Flavonoid biosynthesis’ (ko00941), ‘MAPK signaling pathway—plant’ (ko04016), ‘Cutin, suberine and wax biosynthesis’ (ko00073), ‘Sesquiterpenoid and triterpenoid biosynthesis’ (ko00909), and ‘Stilbenoid, diarylheptanoid and gingerol biosynthesis’ (ko00945).

### 2.5. Secondary Metabolic Pathway Gene Transcript Levels

#### 2.5.1. Differential Genes and Enrichment Pathways Analysis

‘Plant–pathogen interaction’ (ko04626) [30], ‘Phenylpropanoid biosynthesis’ (ko00940) [31], ‘Flavonoid biosynthesis’ (ko00941) [32], ‘MAPK signaling pathway—plant’ (ko04016) [33], and ‘Stilbenoid, diarylheptanoid and gingerol biosynthesis’ (ko00945) [34] are identified and found to be important in plant response to environmental stress. The DEGs were screened according to the criterion of log_2_FPKM ≥ 2. And the analysis revealed that there were 11 differential genes that were enriched in both plant pathogen interactions (ko04626) and signaling factor (ko04016) pathways, and 5 differential genes in phenylpropane biosynthesis, flavonoid biosynthesis, and resveratrol, biphenyl heptanone, and gingerol biosynthetic pathways were co-enriched (Figure S4).

#### 2.5.2. Plant–Pathogen Interaction and MAPK Signaling Pathway—Plant

**Figure 5:**
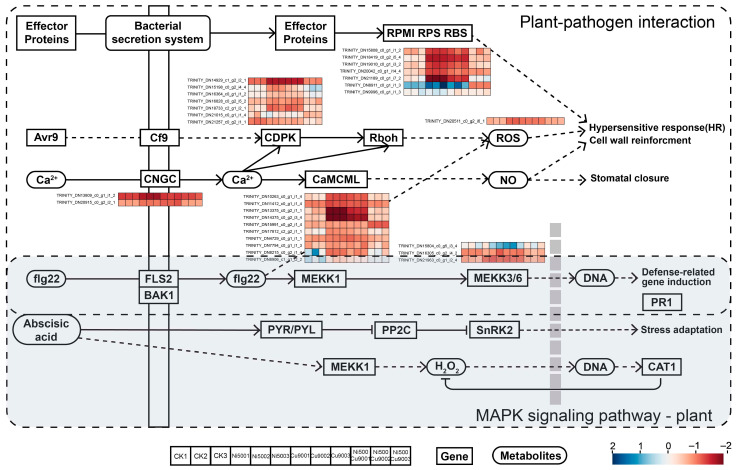
Plant–pathogen interaction and MAPK signaling pathway—plant. Note: RPMI: disease resistance protein RPM1; CDPK: calcium-dependent protein kinase; Rbof: respiratory burst oxidase; CNGC: cyclic nucleotide gated channel, plant; CaMCML: calmodulin.

Following Ni-Cu combined stress, 20 differentially expressed genes (DEGs) were identified in the plant–pathogen interaction pathway and 46 DEGs in the MAPK signaling pathway, with 23 DEGs co-expressed in both pathways. During Ni-Cu single and combined stresses, the plant–pathogen interaction pathway exhibited significant changes in related enzyme genes (Figure 5) such as CNGC, CDPK, Rboh, CaMCML, MPK, PTI, RPMI, RPS, and RBS. In this pathway, after calcium ions entered the cells through CNGC, the genes CDPK, Rboh, and CaMCML were up-regulated in the combined stress group (N5C9), ultimately leading to the generation of reactive oxygen species and nitric oxide (NO), which triggered a defense response. In contrast, bacteria primarily inject effector proteins (EPs) into plant cells through the bacterial secretion system [14,35]. These effector proteins (e.g., AvrPto, AvrPtoB) up-regulate the expression of their corresponding genes, which are recognized by disease resistance proteins (e.g., RPMI, RPS, RBS) in plant cells, triggering defense responses along with ROS [36]. The MAPK signaling pathway is a subclass of signal transduction pathways. Among the aforementioned proteins, CNGC, CDPK, Rbof, and MPK are also significantly enriched in the plant hormone signaling pathway. The expression of PYR, activated by the contrast between Ni-Cu single and complex treatments, is up-regulated, leading to stress adaptation and indirectly influencing CAT-related synthases. In both pathways, FLG enters the cell via FLS in complex with BAK, resulting in the up-regulation of MPK [37], which induces defense-related gene expression (Figure 5).

#### 2.5.3. Phenylpropanoid Biosynthesis, Flavonoid Biosynthesis, and Stilbenoid, Diarylheptanoid, and Gingerol Biosynthesis

**Figure 6:**
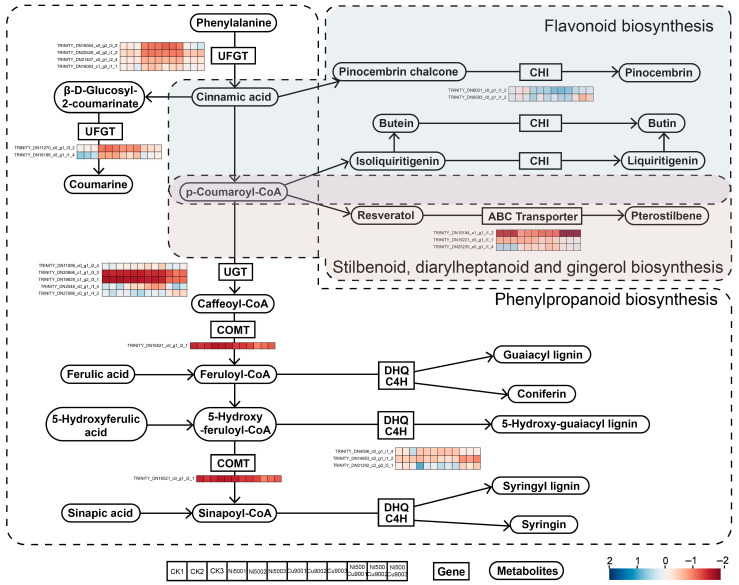
Phenylpropanoid biosynthesis, flavonoid biosynthesis, and stilbenoid, diarylheptanoid, and gingerol biosynthesis. Note: UFGT: anthocyanidin 3-O-glucosyltransferase; UGT: UDP-glucosyltransferase; COMT: caffeic acid O-methyltransferase; C4H: cinnamate 4-hydroxylase; CHI: chalcone isomerase; DHQ: 3-dehydroquinate dehydratase.

A total of 21 DEGs were identified in the phenylpropane biosynthesis pathway, 8 DEGs in the flavonoid biosynthesis pathway, and 9 DEGs in the pathways leading to resveratrol, biphenylheptanone, and gingerol. Five of these DEGs were co-expressed across all three pathways, while one gene was co-expressed in the flavonoid biosynthesis pathway together with the others (Figure S4). In the 3 biosynthetic pathways, genes significantly enriched for UGT, which is involved in the generation of caffeoyl-CoA, were found to be identical (Figure 6).

In the phenylpropane biosynthesis pathway, the genes responsible for synthesizing cinnamic acid and coumarinate from phenylalanine were significantly up-regulated under both Ni-Cu single and combined stress conditions (Figure 6). Phenylpropane metabolism converts simple derivatives (e.g., caffeic acid, ferulic acid) into complex lignin monomers and their glycosides (e.g., guaiacyl lignin, coniferin). DHQ dehydratase and C4H-related genes are differentially expressed during synthesis compared to Ni-Cu single stress. In the flavonoid biosynthetic pathway, pinocembrin chalcone and isoliquiritigenin were synthesized from cinnamic acid and p-coumaroyl-CoA, respectively. Additionally, chalcone isomerase (CHI) expression was elevated in both the combined stress (Ni500+Cu900) and single stress groups (Ni500, Cu900). In the stilbenoid, diarylheptanoid, and gingerol biosynthesis pathway, the genes associated with ABC transporter proteins were significantly more abundant in the combined stress group (Ni500+Cu900) than in the Ni-Cu single stress group (Ni500, Cu900). ABC transporter proteins typically facilitate the transmembrane transport of secondary metabolites rather than participate directly in catalytic reactions.

### 2.6. Correlation Analysis of Physiological Indicators and Genes Under Different Ni-Cu Stresses

**Figure 7:**
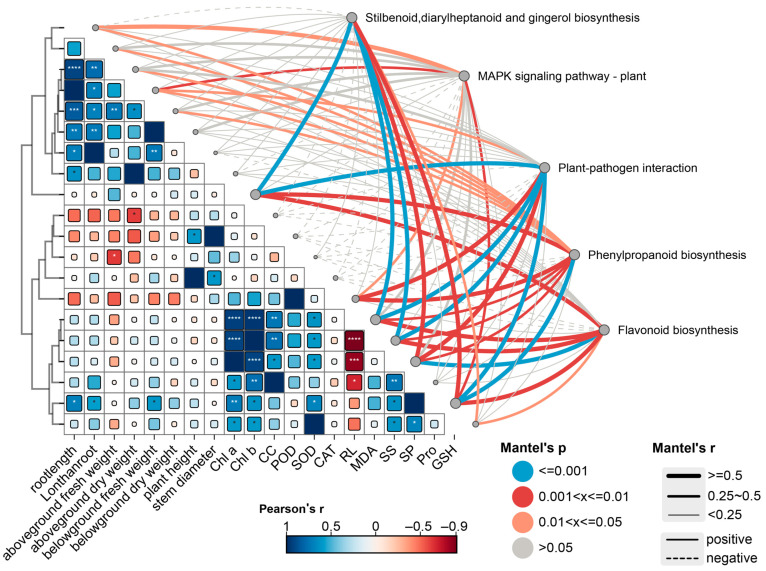
Correlation analysis between physicochemical indexes and gene expression of *E. marginata*. Note: Growth index: root length, lanthan root(root length than weight), aboveground fresh weight; aboveground dry weight; belowground fresh weight; belowground dry weight. Physiological indicators: Chl a, Chl b, CC, POD, SOD, CAT, RL, MDA, SS, SP, Pro, GSH. The lines represent the correlation between related genes and environmental factors, with thicker lines indicating stronger correlations. Solid lines: positive correlations. Dotted lines: negative correlations. *: *p* < 0.05, **: *p* < 0.01, and ***: *p* < 0.005, ****: *p* < 0.001.

Pearson correlation analysis indicated significant correlations between *E. marginata* growth and physiological indices (Figure 7). *E. marginata* root length was significantly and positively correlated with MDA and Pro (*p* < 0.001). Additionally, the content of phyto-photosynthetic pigments (Chl a, Chl b, CC) was positively correlated with osmoregulatory substances (SP, SS) and negatively correlated with cellular membrane permeability indicators, such as relative conductivity (*p* < 0.005) (Table S2).

Mantel test analysis revealed significant correlations between transcribed genes and physicochemical indicators of *E. marginata* (Figure 7). The results indicated that genes differentially expressed in all five enrichment pathways were significantly correlated with the content of plant photosynthetic pigments, relative conductivity, and soluble sugars and proteins (r < 0.9, *p* < 0.005). Additionally, differentially expressed genes in the MAPK signaling pathway and the plant–pathogen interaction pathway correlated with root length (r < 0.6, *p* < 0.05). Flavonoid biosynthesis was correlated with stilbenoid, while differentially expressed genes in the diarylheptanoid and gingerol biosynthesis pathways significantly correlated with SOD (r < 0.6, 0.001 < *p* < 0.005). Genes in the phenylpropanoid biosynthesis pathway significantly correlated with root length, biomass, POD, MDA, and Pro (r < 0.6, 0.001 < *p* < 0.005). The correlation between changes in plant phenotypic and physicochemical indices and differentially expressed genes in *E. marginata* under various treatments indicated that Ni-Cu stress affects the antioxidant and osmoregulatory systems by regulating the transcriptional expression of plant secondary metabolites, thereby conferring resistance to heavy metal stress (Table S3).

## 3. Discussion

It was found that a single Ni or Cu stress significantly affected the growth of *E. marginate* (Figure 1), for example, by suppressing plant height and root length [38]. However, under combined conditions, the coexistence of two heavy metals, Ni and Cu, will mutually inhibit the stress responses of plants to each other. The inhibition of plant height of marginal leaf green in the combined condition group was more severe than that under single Cu stress (decreased by 3.12%), but slightly better than that under single Ni stress (increased by 7.13%). However, the stem diameter of the plants and the biomass of the underground part were similar under single and combined Ni-Cu stress (Ni500, Cu900, and Ni500+Cu900). This is consistent with the results of *Suaeda liaotungensis* under Cu stress [39]. The response of *Tetraena qataranse* to Cu and Ni illustrated that two heavy metals inhibit plant growth [40]. Heavy metals affect cell elongation by inhibiting the formation of cytoskeletal proteins, which may be the reason for the inhibition of seedling growth under heavy metal stress. The accumulation of Ni and Cu by *E. marginata* under stress conditions (Ni500, Cu900, Ni500+Cu900) showed a higher content in the underground part than in the aboveground part, tending to accumulate Ni and Cu in the roots. Not only that, the Cu-Ni combined conditions affect the enrichment of plants for single heavy metal ions. The enrichment characteristics of *E. marginata* are the same as those of most plants and show an exocytosis mechanism like *Aurinia saxatilis* [26,40]. These growth responses and metal accumulation patterns highlight the challenges faced by *E. marginata* under heavy metal stress and provide a basis for further exploration of its physiological responses.

Heavy metal-induced oxidative stress suppresses plant growth, biomass accumulation (Figure 1), and photosynthetic pigment (Figure 2) synthesis [41,42,43]. Plants mitigate this damage through antioxidant synthesis (e.g., CAT, POD, and specialized metabolites) and osmoregulatory compound production to enhance cellular water retention [44,45,46]. Correlation and redundancy analyses showed that heavy metal stress affects plant antioxidant enzyme activity and osmotic regulator content. Under the single stress conditions of Cu and Ni (Ni500, Cu900), SOD and POD activities all showed a significantly higher trend compared to the CK group, with the increase in POD activity being significantly higher than that of CAT, implying that POD may play a dominant role in the process of scavenging H_2_O_2_ [47,48,49]. In contrast, the combined Ni-Cu stress, compared with copper stress, reduced the POD and CAT activity and increased the MDA content simultaneously, indicating the antagonistic metal interaction. These likely stem from competitive ion uptake inhibition causing ROS bursts [50,51]. In addition, it was found that under single stress of Cu or Ni, the levels of Pro and SP were significantly lower than in the CK group. However, both showed a decreasing trend in combined stress. The results of this study align with findings that *Theobroma cacao* [52] increases osmotic protectants in response to Ni toxicity, a phenomenon also described in *Zea mays* [53]. However, excessive stress concentrations overwhelm proline’s regulatory capacity, inducing irreversible membrane lipid peroxidation and systemic collapse of antioxidant/osmoregulatory defenses [54]. These findings establish a framework for elucidating Ni-Cu resistance mechanisms of *E. marginata* through integrated gene expression profiling and physiological indicator analysis.

Under Ni-Cu single or combined stress, calmodulin (CaM) expression significantly changed in single treatments (Ni500, Cu900) but restored to near-control levels under combined stress (Ni500+Cu900) (Figure 5). Suppressed CaM expression under combined stress correlated with reduced antioxidant enzyme activities, indicating exceeded oxidative stress thresholds [55]. MAPK cascade components (e.g., MKK3/6) exhibited differential expression under single Ni or Cu stresses but decreased under combined stress [56]. ROS-activated kinases exacerbate oxidative damage [57,58,59]. Combined stress may saturate Ca^2+^-CaM buffering, disrupting MAPK cascades and aligning with ROS-MAPK synergy beyond stress thresholds [60]. Flavonoid antioxidants contribute to stress resistance (Figure 6). PAL and C4H catalyze initial steps of phenylpropanoid metabolism to generate p-coumaric acid [61,62,63]. UFGT expression (critical for coumarin synthesis [63]) increased under single Ni or Cu stress but decreased under combined treatment. Heavy metal ions may activate enzymes synthesizing p-coumaroyl-CoA, enhancing molecular stability/activity. Flavonoids scavenge H_2_O_2_ via phenolic acid/ascorbate-peroxidase cycles [64]. DEG enrichment highlighted phenylpropanoid biosynthesis (ko00940) and MAPK signaling (ko04016) as predominant. Phenylalanine-derived pathways (ko00941, ko00945) were significantly enriched. Studies on *Zingiber officinale* [65,66] and *Miscanthus lutarioriparius* [67] under stress confirm the critical role of these phenylpropanoid-derived pathways in heavy metal resistance.

## 4. Materials and Methods

### 4.1. Plant Material and Growing Conditions

*E. marginata* were provided by the Changjing Nursery in Shuyang County, and the experiments were conducted at the experimental base of Jilin Agricultural University. Healthy, plump seeds were selected and then soaked in 0.5% KMnO_4_ for 3 min to disinfect the surface. The seeds were thoroughly washed with deionized water. The seeds were sown in seedling trays and transplanted into pots (with a wide diameter) once the plants developed 3–4 true leaves. 3 seedlings were planted in each pot, using garden soil as the growing medium. The potted plants were placed in an artificial greenhouse at the experimental base, where the daytime temperature was maintained at 26 ± 1 °C, the nighttime temperature at 20 ± 1 °C, with a controlled photoperiod of 8 h light (5000–12,000 lux) and 16 h dark daily, and the humidity ranged from 50% to 70%. Watering was performed daily to maintain soil moisture.

### 4.2. Experimental Designs

After allowing *E. marginata* seedlings to acclimate in pots for 7 days, they were divided into 4 groups. Each group underwent 3 biological replications. Distilled water was used as the control (CK), and 0.6 L of the heavy metal solution was placed in the flowerpot. The solution concentrations of different treatment groups were 900 mg/kg of Cu (Cu900), 500 mg/kg of Ni (Ni500), and 900 mg/kg of Cu and 500 mg/kg of Ni (Ni500+Cu900). Ni^2+^ and Cu^2+^ solutions were prepared from analytically pure NiCl_2_·6H_2_O and CuSO_4_·5H_2_O, respectively [67]. Morphological and physiological indices were assessed after 45 days. Some samples were frozen in liquid nitrogen and stored at −80 °C for transcriptomic sequencing.

### 4.3. Indicator Measurement

#### 4.3.1. Measurement of Growth Indicators

45 days after the application of heavy metal stress, *E. marginata* plants with relatively uniform growth from each group (CK, Ni500, Cu900, Ni500+Cu900) were selected and divided into aboveground and underground parts at the root neck. The aboveground parts were measured for plant height using a tape measure, while stem diameter was measured 2 mm above the ground using a vernier caliper. The underground parts were washed and scanned morphologically using an EPSON Expression 12000XL (Epson, Suwa, Japan). Root length, projected area, surface area, volume, and the number of root tips were measured using WinRHIZO 2022 root measurement and analysis software. The fresh weights of the aboveground and belowground parts of the plants were measured using a balance (Lichen, Shanghai, China). They were subsequently placed in a cool area to air dry until reaching a constant weight, after which the corresponding dry weights were measured, and specific root weights were calculated.

#### 4.3.2. Measurement of Physiological Indicators

Chlorophyll content was measured via ethanol-acetone immersion. Fresh leaf samples (1 g) were immersed in 10 mL of 95% ethanol:80% acetone (1:1, *v*/*v*) and stored in darkness. Chlorophyll a and b concentrations were calculated from absorbance values at 665 nm, 649 nm and 470 nm using a spectrophotometer. C_a_, C_b_, and C_c_ respectively represent the concentrations of chlorophyll a, chlorophyll b, and carotenoids. A_665_, A_649_, and A_470_ respectively represent the absorbance of the extract at 665 nm, 649 nm, and 470 nm.

C_a_ = 13.95 A_665_ − 6.88 A_649_ (1)

C_b_ = 24.96 A_649_ − 7.32 A_665_ (2)

C_c_ = (1000 A_470_ − 2.05 C_a_ − 114 C_b_)/245 (3)

The SOD activity was determined through the nitrogen blue tetrazolium method [68]. Frozen leaves (0.1 g) were homogenized in 10 mL of 0.01 mol/L phosphate buffer (pH 6.0) on ice. After centrifugation (4000 rpm, 15 min), 1 mL of supernatant was mixed with 1 mL of buffer as CK. A 3 mL volume of the reaction mixture was added, and absorbance at 470 nm was recorded at 1 min intervals (5 readings total).

The POD activity was determined through the guaiacol color development method [68]. Fresh leaves (1 g) were homogenized in 10 mL of phosphate buffer and centrifuged (4000 rpm, 15 min). Then 1 mL of supernatant was added to 3 mL of the reaction mixture. Absorbance at 470 nm was measured every minute with a spectrophotometer (Shanghai Jingke, Shanghai, China). The reaction mixture consisted of potassium phosphate buffer, hydrogen peroxide, and guaiacol.

The CAT activity was determined by the ultraviolet absorption method [68]. Frozen leaves (0.1 g) were homogenized in 5 mL ice-cold phosphate buffer (0.1 mol/L, pH 7.5) containing 5 mmol/L DTT and 5% PVP. After centrifugation (12,000 rpm, 10 min, 4 °C), 100 μL of supernatant was mixed with 2.9 mL of 0.02 mol/L H_2_O_2_. Absorbance at 240 nm was measured at 1 min intervals (5 readings). CAT activity was determined by referencing the standard linear equation and the change in absorbance value.

The content of malondialdehyde (MDA) was determined by the thiobarbituric acid method [69]. Frozen leaves (0.1 g) were ground with 5 mL of ice-cold 5% TCA. After centrifugation (4000 rpm, 10 min), 2 mL of supernatant was collected. For the CK, 2 mL of distilled water was used. Subsequently, 2 mL of 0.6% TBA was added to the solutions. The mixtures were boiled in a water bath for 10 min, then cooled and centrifuged at 3000 rpm for 15 min. MDA content was determined by measuring supernatant absorbance at 532 nm and 600 nm.

The relative conductivity was determined using the immersion method [69]. Fresh leaf segments (0.1 g) were immersed in 20 mL of distilled water for 24 h at room temperature. The conductivity of the extract was measured using a conductivity meter (INASE Scientific Instrument Co., Ltd., Shanghai, China). The extract was then heated in a boiling water bath for 15 min, and the conductivity was measured again after cooling. The relative conductivity values were then calculated.

The soluble protein content was measured using the Coomassie brilliant blue G-250 staining method [68]. Fresh leaves (1 g) were homogenized in 5 mL of phosphate buffer and centrifuged (4000 rpm, 10 min). Next, we added the supernatant to the Coomassie brilliant blue solution and performed colorimetry at 595 nm with the help of a spectrophotometer (Shanghai Jingke, Shanghai, China). Finally, we calculated the soluble protein content based on the standard curve.

The content of soluble sugar (SS) was determined by the anthrone method [69]. Approximately 0.1 g of liquid-nitrogen-frozen leaves were homogenized in 1.5 mL of 80% ethanol in an ice bath. The mixture was transferred to a 50 °C water bath and incubated for 20 min, with mixing every 2 min. After cooling, the mixture was centrifuged at 12,000 rpm for 10 min at room temperature. Then 25 μL of supernatant was mixed with 75 μL of water, 30 μL of anthrone reagent, and 250 μL of concentrated H_2_SO_4_. The mixtures were then heated in a water bath at 95 °C for 10 min. The absorbance was measured at 620 nm, and the soluble sugar content was calculated using the standard linear equation.

Proline content was determined using ninhydrin colorimetry [69]. Frozen leaves (0.1 g) were homogenized in 1 mL ice-cold 3% sulfosalicylic acid. The homogenate was heated at 90 °C for 10 min with shaking, then centrifuged (12,000 rpm, 10 min). The supernatant was cooled. Then 150 μL of cooled supernatant was mixed with 150 μL of distilled water, 150 μL of glacial acetic acid, and 300 μL of 3% sulfosalicylic acid. The mixture was heated at 95 °C for 30 min. Absorbance at 520 nm was measured after cooling, and proline content was calculated from a standard curve.

GSH content was determined using the DTNB method [70]. Frozen leaves (0.1 g) were homogenized in 1 mL of ice-cold 3% sulfosalicylic acid. Centrifuge at 12,000 rpm for 10 min at 4 °C, then cool the supernatant. Then 20 μL of supernatant was mixed with 120 μL of phosphate buffer (0.1 mol/L, pH 8.0) and 40 μL of DTNB reagent. After 5 min incubation, absorbance at 412 nm was measured, and the GSH content was calculated using the standard linear equation.

All of the reagents mentioned above were purchased from Changchun Anmei Biotechnology Co. (Changchun, China).

#### 4.3.3. Determination of Heavy Metal Content

The plants of *E. marginata* were washed with distilled water for 45 d, the surface water was blotted with filter paper, and 0.2 g each of the roots, stems, and leaves were weighed, and the heavy metal contents were determined by microwave digestion of the samples using inductively coupled plasma mass spectrometry (ICP-MS) according to the National Standard of China for Food Safety for the Determination of Multiple Elements in Food (GB 5009.268-2016); the soil treated for 45 d was dried in a constant-temperature drying oven at 37 °C until constant weight, then ground in a mortar and passed through a 100-mesh sieve, and the heavy metal contents were determined in the same way. After removing the residual plant tissues, the soil was dried to constant weight at 37 °C in a constant temperature drying oven and then ground in a mortar and passed through a 100-mesh sieve, and then the heavy metal content was determined in the same way as above.

#### 4.3.4. Transcriptomics Analysis

Samples were divided into 4 groups: CK, Ni500, Cu900, and Ni500+Cu900, each with three biological replicates. Before RNA extraction, samples were rinsed with deionized water, blotted dry, flash-frozen in liquid nitrogen, and stored at −80 °C. Total RNA was isolated using TRIzol reagent following the manufacturer’s protocol. RNA purity and concentration were assessed using a NanoDrop 2000 spectrophotometer (Thermo Scientific, Waltham, MA, USA), while RNA integrity was evaluated using an Agilent 2100 Bioanalyzer (Agilent Technologies, Santa Clara, CA, USA), with RNA Integrity Numbers (RIN) > 8.0. Strand-specific RNA-seq libraries (insert size: 150–250 bp) were prepared using the VAHTS Universal V6 RNA-seq Library Prep Kit (Vazyme Biotech, Nanjing, China, CN61002) in strict accordance with the Illumina TruSeq library (Illumina, San Diego, CA, USA) construction protocol. Library quality was verified using an Agilent 4200 TapeStation, confirming a primary fragment distribution of 145–220 bp and a concentration > 2 nM. Sequencing was performed on an Illumina NovaSeq 6000 platform (PE150 mode). Transcriptome sequencing and bioinformatic analyses were conducted by Shanghai OE Biotech Co., Ltd. (Shanghai, China).

Raw sequencing reads were quality-filtered using Trimmomatic to remove poly-N-containing reads and low-quality sequences (Q < 20), yielding clean reads. De novo assembly of clean reads was performed using Trinity to generate transcript isoforms. The longest transcripts with >95% sequence similarity were selected as unigenes. Functional annotation was conducted by aligning unigenes against the NCBI NR, Swiss-Prot, eggNOG, and KOG databases using Diamond (E-value < 1 × 10^−5^). Pathway annotation was performed via KEGG, while GO classification was derived from Swiss-Prot associations. Read counts were mapped using Bowtie, and gene expression levels (FPKM) were quantified with eXpress. Differential expression analysis was performed using DESeq2, applying a negative binomial distribution test. Differentially expressed genes (DEGs) were identified under the threshold of *q* < 0.05 and |FoldChange| > 2. Hierarchical clustering of DEGs across experimental groups was visualized using R (v3.2.0). Enrichment analysis of GO terms and KEGG pathways was conducted via hypergeometric testing, and statistically significant terms (*q* < 0.05) were graphically represented.

The transcript abundance of the 5 DEGs was assessed by RT-qPCR analysis, which verified the accuracy and reproducibility of the transcriptome data. The expression trends in the RNA-seq data were consistent with the qPCR gene expression patterns (Figure S5), demonstrating that our RNA-seq data are highly reliable, reproducible, and accurate.

### 4.4. Data Analysis

Data were collated and computed using Microsoft Excel 2024. IBM SPSS Statistics 25.0 (SPSS, Inc., Chicago, IL, USA) was used to perform one-way ANOVA and interaction analysis between samples, Duncan’s method was used to test for variability between treatments and Pearson correlation analysis, Canoco5 was used for redundancy analysis, R-4.5.3 was used for interaction effect analysis, and Origin 2024 was used for plotting with R; data graphs were plotted as mean ± standard error (mean ± SE) expressing relative change calculated as (mean treatment group − mean CK group)/mean CK group.

## 5. Conclusions

This study revealed the physiological and molecular synergistic regulatory mechanisms of *E. marginata* in response to single and combined Ni-Cu stress. Under single stress (Ni^2+^ or Cu^2+^), plants alleviated oxidative damage by significantly enhancing the activities of antioxidant enzymes and up-regulating osmoregulatory, with the dominant role of POD on H_2_O_2_ scavenging highlighting its core compensatory function. However, the reduced activities of antioxidant enzymes and decreased MDA content under combined stress indicated an antagonistic effect of Cu^2+^ and Ni^2+^, leading to an overshooting of the oxidative stress threshold and exacerbating membrane lipid peroxidation. In terms of molecular mechanisms, calmodulin (CaM)-related genes were differentially expressed under Ni or Cu single stress and restored to the CK level in combined stress; presumably, metal ions competitively interfered with Ca^2+^ homeostasis, disrupted Ca^2+^-CaM signaling, and indirectly triggered the ROS outbreak; the MAPK signaling pathway homogenized response further confirmed the positive feedback regulation of the ROS-MAPK cascade. Phenylpropane-derived metabolism pathways were significantly enriched, suggesting that flavonoid combinations may enhance tolerance by chelating metals or scavenging ROS. This study provides a key theoretical basis for resolving the synergistic defenses strategy of complex heavy metal stress in plants. The article merely explored the differential expression of some genes, but this does not imply any changes in the content of related proteins. It is hoped that future research can supplement the differential expression of related metabolites and explain the correlation between gene differential expression and differential metabolites.

## Data Availability

The original contributions presented in this study are included in the article. Further inquiries can be directed to the corresponding authors.

## Supplementary Materials

The following supporting information can be downloaded at https://www.mdpi.com/article/10.3390/plants14142159/s1: Figure S1: Correlation analysis between different samples; Figure S2. Volcano map of differential genes under Ni and combined stresses; Figure S3. Volcano map of differential genes under Cu and combined stresses; Figure S4. Venn diagram of differential gene expression versus gene number in five enrichment pathways; Figure S5. Validation of RNA-seq data by qRT-PCR showing concordant expression trends of 5 differentially expressed genes; Table S1. Interaction analysis of various indexes of *E. marginata* under combined Cu-Ni stresses; Table S2. Pearson’s correlation analysis of growth and physiological indicators of *E. marginata*; Table S3. Mantel test analysis of physiological indicators and genes under different Cu-Ni stresses. Table S4. Under single and combined conditions of Ni and Cu, the heavy metal contentin in soil and various parts of and plants, enrichment coefficient and transport coefficient. Table S5. RDA.

## References

[1] Pilon-Smits E. (2005). Phytoremediation. Annu. Rev. Plant Biol..

[2] Kim Y.-O., Safdar M., Kang H., Kim J. (2024). Glycine-Rich RNA-Binding Protein AtGRP7 Functions in Nickel and Lead Tolerance in Arabidopsis. Plants.

[3] Elshkaki A., Reck B.K., Graedel T.E. (2017). Anthropogenic nickel supply, demand, and associated energy and water use. Resour. Conserv. Recycl..

[4] Liu L., Zhang L., Jiang S., Yuan Z., Chen J. (2023). Global copper cycles in the anthroposphere since the 1960s. Resour. Conserv. Recycl..

[5] Zhao J., Xu Z., Wang X., Wan S., Chen W., Huang W., Wang M., Wang R., Zhang H. (2024). Environmental copper exposure, placental cuproptosis, and miscarriage. Environ. Pollut..

[6] Chen L., Zhou M., Wang J., Zhang Z., Duan C., Wang X., Zhao S., Bai X., Li Z., Li Z. (2022). A global meta-analysis of heavy metal(loid)s pollution in soils near copper mines: Evaluation of pollution level and probabilistic health risks. Sci. Total Environ..

[7] Yan K., Wang H., Lan Z., Zhou J., Fu H., Wu L., Xu J. (2022). Heavy metal pollution in the soil of contaminated sites in China: Research status and pollution assessment over the past two decades. J. Clean. Prod..

[8] Khan D., Yang X., He G., Khan R.A.A., Usman B., Hui L., Khokhar A.A., Zaman Q.U., Wang H.-F. (2024). Comparative Physiological and Transcriptomics Profiling Provides Integrated Insight into Melatonin Mediated Salt and Copper Stress Tolerance in *Selenicereus undatus* L. *Plants*
**2024**, *13*, 3602. Plants.

[9] Saleh M.A. (2024). Nickel toxicity mitigation by supplementation of acetylcholine in wheat: Growth, photosynthetic and antioxidant activities. S. Afr. J. Bot..

[10] Qin C., Shen J., Ahanger M.A. (2022). Supplementation of nitric oxide and spermidine alleviates the nickel stress-induced damage to growth, chlorophyll metabolism, and photosynthesis by upregulating ascorbate–glutathione and glyoxalase cycle functioning in tomato. Front. Plant Sci..

[11] Soliman M., Alhaithloul H.A., Hakeem K.R., Alharbi B.M., El-Esawi M., Elkelish A. (2019). Exogenous Nitric Oxide Mitigates Nickel-Induced Oxidative Damage in Eggplant by Upregulating Antioxidants, Osmolyte Metabolism, and Glyoxalase Systems. Plants.

[12] Marastoni L., Tauber P., Pii Y., Valentinuzzi F., Astolfi S., Simoni A., Brunetto G., Cesco S., Mimmo T. (2019). The potential of two different *Avena sativa* L. cultivars to alleviate Cu toxicity. Ecotoxicol. Environ. Saf..

[13] Shabbir Z., Sardar A., Shabbir A., Abbas G., Shamshad S., Khalid S., Natasha, Murtaza G., Dumat C., Shahid M. (2020). Copper uptake, essentiality, toxicity, detoxification and risk assessment in soil-plant environment. Chemosphere.

[14] Crizel R.L., Perin E.C., Vighi I.L., Woloski R., Seixas A., da Silva Pinto L., Rombaldi C.V., Galli V. (2020). Genome-wide identification, and characterization of the CDPK gene family reveal their involvement in abiotic stress response in *Fragaria* x *ananassa*. Sci. Rep..

[15] Ahmad P., Ahanger M.A., Alyemeni M.N., Wijaya L., Alam P. (2017). Exogenous application of nitric oxide modulates osmolyte metabolism, antioxidants, enzymes of ascorbate-glutathione cycle and promotes growth under cadmium stress in tomato. Protoplasma.

[16] Steinhorst L., Kudla J. (2014). Signaling in cells and organisms—Calcium holds the line. Curr. Opin. Plant Biol..

[17] Yue Z., Chen Y., Chen C., Ma K., Tian E., Wang Y., Liu H., Sun Z. (2021). Endophytic Bacillus altitudinis WR10 alleviates Cu toxicity in wheat by augmenting reactive oxygen species scavenging and phenylpropanoid biosynthesis. J. Hazard. Mater..

[18] Kaur R., Yadav P., Sharma A., Kumar Thukral A., Kumar V., Kaur Kohli S., Bhardwaj R. (2017). Castasterone and citric acid treatment restores photosynthetic attributes in *Brassica juncea* L. under Cd(II) toxicity. Ecotoxicol. Environ. Saf..

[19] An Q., Wen C., Yan C. (2024). Meta-analysis reveals the combined effects of microplastics and heavy metal on plants. J. Hazard. Mater..

[20] Goff J.L., Chen Y., Thorgersen M.P., Hoang L.T., Poole F.L., Szink E.G., Siuzdak G., Petzold C.J., Adams M.W.W. (2023). Mixed heavy metal stress induces global iron starvation response. ISME J..

[21] Ahmad J., Qamar S., Nida, Khan F., Haq I., Al-Huqail A., Qureshi M.I. (2020). Differential impact of some metal(loid)s on oxidative stress, antioxidant system, sulfur compounds, and protein profile of Indian mustard (*Brassica juncea* L.). Protoplasma.

[22] Cao D.-j., Xie P.-p., Deng J.-w., Zhang H.-m., Ma R.-x., Liu C., Liu R.-j., Liang Y.-g., Li H., Shi X.-d. (2015). Effects of Cu^2+^ and Zn^2+^ on growth and physiological characteristics of green algae, Cladophora. Environ. Sci. Pollut. Res..

[23] Zhang H.Z., Li H., Wang Z., Zhou L.D. (2011). Accumulation Characteristics of Copper and Cadmium in Greenhouse Vegetable Soils in Tongzhou District of Beijing. Procedia Environ. Sci..

[24] Xu L., Xing X., Peng J., Ji M., Sovago I. (2022). Estimation of Copper and Cadmium Bioavailability in Contaminated Soil Remediated by Different Plants and Micron Hydroxyapatite. Bioinorg. Chem. Appl..

[25] Moyne A.L., Souq F., Yean L.H., Brown S.C., Boulay M., Sangwan-Norreel B.S. (1993). Relationship between cell ploidy and regeneration capacity of long term *Rosa hybrida* cultures. Plant Sci..

[26] Zhang X., Cui W., Yan J., Yang X., Chen M., Jiang P., Yu G. (2025). Physiological responses of *Leersia hexandra* Swart to Cu and Ni Co-contamination: Implications for phytoremediation. Environ. Technol. Innov..

[27] Chen M., Jiang P., Zhang X., Sunahara G.I., Liu J., Yu G. (2024). Physiological and biochemical responses of *Leersia hexandra* Swartz to nickel stress: Insights into antioxidant defense mechanisms and metal detoxification strategies. J. Hazard. Mater..

[28] He G., Xie H., Tan B., Chen M., Wu Z., Dai Z., Sun R., He L., Li C. (2025). Effects of microplastics and heavy metal stress on the growth and physiological characteristics of pioneer plant *Avicennia marina*. Mar. Pollut. Bull..

[29] Liang J., Wang Z., Ren Y., Jiang Z., Chen H., Hu W., Tang M. (2023). The alleviation mechanisms of cadmium toxicity in *Broussonetia papyrifera* by arbuscular mycorrhizal symbiosis varied with different levels of cadmium stress. J. Hazard. Mater..

[30] Zarattini M., Farjad M., Launay A., Cannella D., Soulié M.-C., Bernacchia G., Fagard M., Noctor G. (2021). Every cloud has a silver lining: How abiotic stresses affect gene expression in plant-pathogen interactions. J. Exp. Bot..

[31] Wang Z., Yang J., Gao Q., He S., Xu Y., Luo Z., Liu P., Wu M., Xu X., Ma L. (2023). The transcription factor NtERF13a enhances abiotic stress tolerance and phenylpropanoid compounds biosynthesis in tobacco. Plant Sci..

[32] Su L., Lv A., Wen W., Fan N., Li J., Gao L., Zhou P., An Y. (2022). MsMYB741 is involved in alfalfa resistance to aluminum stress by regulating flavonoid biosynthesis. Plant J..

[33] Raja V., Majeed U., Kang H., Andrabi K.I., John R. (2017). Abiotic stress: Interplay between ROS, hormones and MAPKs. Environ. Exp. Bot..

[34] Zhao M., Ren Y., Li Z. (2021). Transcriptome profiling of Jerusalem artichoke seedlings (*Helianthus tuberosus* L.) under polyethylene glycol-simulated drought stress. Ind. Crops Prod..

[35] Li S., Wang Y., Gao X., Lan J., Fu B. (2022). Comparative Physiological and Transcriptome Analysis Reveal the Molecular Mechanism of Melatonin in Regulating Salt Tolerance in Alfalfa (*Medicago sativa* L.). Front. Plant Sci..

[36] Xiong L., Yang Y. (2003). Disease Resistance and Abiotic Stress Tolerance in Rice Are Inversely Modulated by an Abscisic Acid–Inducible Mitogen-Activated Protein Kinase. Plant Cell.

[37] Kim S.H., Bahk S., Nguyen N.T., Pham M.L.A., Kadam U.S., Hong J.C., Chung W.S. (2022). Phosphorylation of the auxin signaling transcriptional repressor IAA15 by MPKs is required for the suppression of root development under drought stress in Arabidopsis. Nucleic Acids Res..

[38] Zhang F.-Q., Wang Y.-S., Lou Z.-P., Dong J.-D. (2007). Effect of heavy metal stress on antioxidative enzymes and lipid peroxidation in leaves and roots of two mangrove plant seedlings (*Kandelia candel* and *Bruguiera gymnorrhiza*). Chemosphere.

[39] Song J., Cao X., An R., Ding H., Wang W., Zhou Y., Wu C., Cao Y., Wang H., Li C. (2025). Physiological Adaptation to Different Heavy Metal Stress in Seedlings of Halophyte *Suaeda liaotungensis*. Biology.

[40] Usman K., Al-Ghouti M.A., Abu-Dieyeh M.H. (2019). The assessment of cadmium, chromium, copper, and nickel tolerance and bioaccumulation by shrub plant *Tetraena qataranse*. Sci. Rep..

[41] Téllez Vargas J., Rodríguez-Monroy M., López Meyer M., Montes-Belmont R., Sepúlveda-Jiménez G. (2017). *Trichoderma asperellum* ameliorates phytotoxic effects of copper in onion (*Allium cepa* L.). Environ. Exp. Bot..

[42] Zaheer I.E., Ali S., Rizwan M., Farid M., Shakoor M.B., Gill R.A., Najeeb U., Iqbal N., Ahmad R. (2015). Citric acid assisted phytoremediation of copper by *Brassica napus* L. *Ecotoxicol*. Environ. Saf..

[43] Habiba U., Ali S., Farid M., Shakoor M.B., Rizwan M., Ibrahim M., Abbasi G.H., Hayat T., Ali B. (2014). EDTA enhanced plant growth, antioxidant defense system, and phytoextraction of copper by *Brassica napus* L. *Environ*. Sci. Pollut. Res..

[44] Rehman M., Liu L., Bashir S., Saleem M.H., Chen C., Peng D., Siddique K.H.M. (2019). Influence of rice straw biochar on growth, antioxidant capacity and copper uptake in ramie (*Boehmeria nivea* L.) grown as forage in aged copper-contaminated soil. Plant Physiol. Biochem..

[45] Yu B., Chao D.Y., Zhao Y. (2024). How plants sense and respond to osmotic stress. J. Integr. Plant Biol..

[46] Soares C., de Sousa A., Pinto A., Azenha M., Teixeira J., Azevedo R.A., Fidalgo F. (2016). Effect of 24-epibrassinolide on ROS content, antioxidant system, lipid peroxidation and Ni uptake in *Solanum nigrum* L. under Ni stress. Environ. Exp. Bot..

[47] Yuce M., Ekinci M., Turan M., Agar G., Aydin M., Ilhan E., Yildirim E. (2024). Chrysin mitigates copper stress by regulating antioxidant enzymes activity, plant nutrient and phytohormones content in pepper. Sci. Hortic..

[48] Shaheen S., Ahmad R., Mahmood Q., Pervez A., Maroof Shah M., Hafeez F. (2019). Gene expression and biochemical response of giant reed under Ni and Cu stress. Int. J. Phytoremediation.

[49] Shang C., Zhou Q., Nkoh J.N., Liu J., Wang J., Hu Z., Hussain Q. (2024). Integrated physiological, biochemical, and transcriptomic analyses of *Bruguiera gymnorhiza* leaves under long-term copper stress: Stomatal size, wax crystals and composition. Ecotoxicol. Environ. Saf..

[50] Hawco N.J., Saito M.A. (2018). Competitive inhibition of cobalt uptake by zinc and manganese in a pacific *Prochlorococcus* strain: Insights into metal homeostasis in a streamlined oligotrophic cyanobacterium. Limnol. Oceanogr..

[51] Szabados L., Savouré A. (2010). Proline: A multifunctional amino acid. Trends Plant Sci..

[52] dos Santos Silva J.V., Baligar V.C., Ahnert D., Pirovani C.P., Mora-Ocampo I.Y., de Vasconcelos L.M., de Almeida A.-A.F. (2025). Proteomic and transcriptional regulations in *Theobroma cacao* L., in response to Ni toxicity, reveal temporal and metabolic reprogramming. J. Hazard. Mater..

[53] Sirhindi G., Mir M.A., Abd-Allah E.F., Ahmad P., Gucel S. (2016). Jasmonic Acid Modulates the Physio-Biochemical Attributes, Antioxidant Enzyme Activity, and Gene Expression in Glycine max Under Nickel Toxicity. Front. Plant Sci..

[54] Lin Y.-J., Yao B.-T., Zhang Q., Feng Y.-X., Xiang L. (2024). Biochemical insights into proline metabolism and its contribution to the endurant cell wall structure under metal stress. Ecotoxicol. Environ. Saf..

[55] Jalmi S.K., Bhagat P.K., Verma D., Noryang S., Tayyeba S., Singh K., Sharma D., Sinha A.K. (2018). Traversing the Links Between Heavy Metal Stress and Plant Signaling. Front. Plant Sci..

[56] Liu H., Li X., He F., Li M., Zi Y., Long R., Zhao G., Zhu L., Hong L., Wang S. (2024). Genome-wide identification and analysis of abiotic stress responsiveness of the mitogen-activated protein kinase gene family in *Medicago sativa* L.. BMC Plant Biol..

[57] Manna M., Rengasamy B., Sinha A.K. (2023). Revisiting the role of MAPK signalling pathway in plants and its manipulation for crop improvement. Plant Cell Environ..

[58] Pitzschke A., Djamei A., Bitton F., Hirt H. (2009). A Major Role of the MEKK1–MKK1/2–MPK4 Pathway in ROS Signalling. Mol. Plant.

[59] Kovtun Y., Chiu W.-L., Tena G., Sheen J. (2000). Functional analysis of oxidative stress-activated mitogen-activated protein kinase cascade in plants. Proc. Natl. Acad. Sci. USA.

[60] Zeng H., Zhu Q., Yuan P., Yan Y., Yi K., Du L. (2023). Calmodulin and calmodulin-like protein-mediated plant responses to biotic stresses. Plant Cell Environ..

[61] Yang J., Ji L., Liu S., Jing P., Hu J., Jin D., Wang L., Xie G., Nakamura Y. (2021). The CaM1-associated CCaMK–MKK1/6 cascade positively affects lateral root growth via auxin signaling under salt stress in rice. J. Exp. Bot..

[62] Vogt T. (2010). Phenylpropanoid Biosynthesis. Mol. Plant.

[63] Dong N.Q., Lin H.X. (2021). Contribution of phenylpropanoid metabolism to plant development and plant–environment interactions. J. Integr. Plant Biol..

[64] Rao M.J., Zheng B. (2025). The Role of Polyphenols in Abiotic Stress Tolerance and Their Antioxidant Properties to Scavenge Reactive Oxygen Species and Free Radicals. Antioxidants.

[65] Jiang Y., Huang M., Wisniewski M., Li H., Zhang M., Tao X., Liu Y., Zou Y. (2018). Transcriptome Analysis Provides Insights into Gingerol Biosynthesis in Ginger (*Zingiber officinale*). Plant Genome.

[66] Chen Z., Zhang L., Lv Y., Qu S., Liu W., Wang K., Gao S., Zhu F., Cao B., Xu K. (2024). A genome assembly of ginger (*Zingiber officinale* Roscoe) provides insights into genome evolution and 6-gingerol biosynthesis. Plant J..

[67] Wang J., Duan X., Wang Y., Sheng J. (2022). Transcriptomic and physiological analyses of *Miscanthus lutarioriparius* in response to plumbum stress. Ind. Crops Prod..

[68] Jiang L., Yun M., Ma Y., Qu T. (2024). Melatonin Mitigates Water Deficit Stress in *Cenchrus alopecuroides* (L.) Thunb through Up-Regulating Gene Expression Related to the Photosynthetic Rate, Flavonoid Synthesis, and the Assimilatory Sulfate Reduction Pathway. Plants.

[69] Zhou X., An Y., Qu T., Jin T., Zhao L., Guo H., Wang W., Zhao C. (2024). Effects of Ni and Cu Stresses on Morphological and Physiological Characteristics of *Euphorbia marginata* Pursh Seedlings. Agronomy.

[70] Griffith O.W. (1980). Determination of glutathione and glutathione disulfide using glutathione reductase and 2-vinylpyridine. Anal. Biochem..

