# Supplementary material for: Euphorbia marginata Alleviate Heavy Metal Ni-Cu Combined Stress by Regulating the Synthesis of Signaling Factors and Flavonoid Organisms
Source: Plants (Basel). 2025 Jul 13;14(14):2159. doi: 10.3390/plants14142159 (PMC12298763; doi:10.3390/plants14142159)
Supplement: Supplementary file 1 [file plants-14-02159-s001.zip › Figure S4. Venn diagram of differential gene expression versus gene number in five enrichment pathways.pdf]

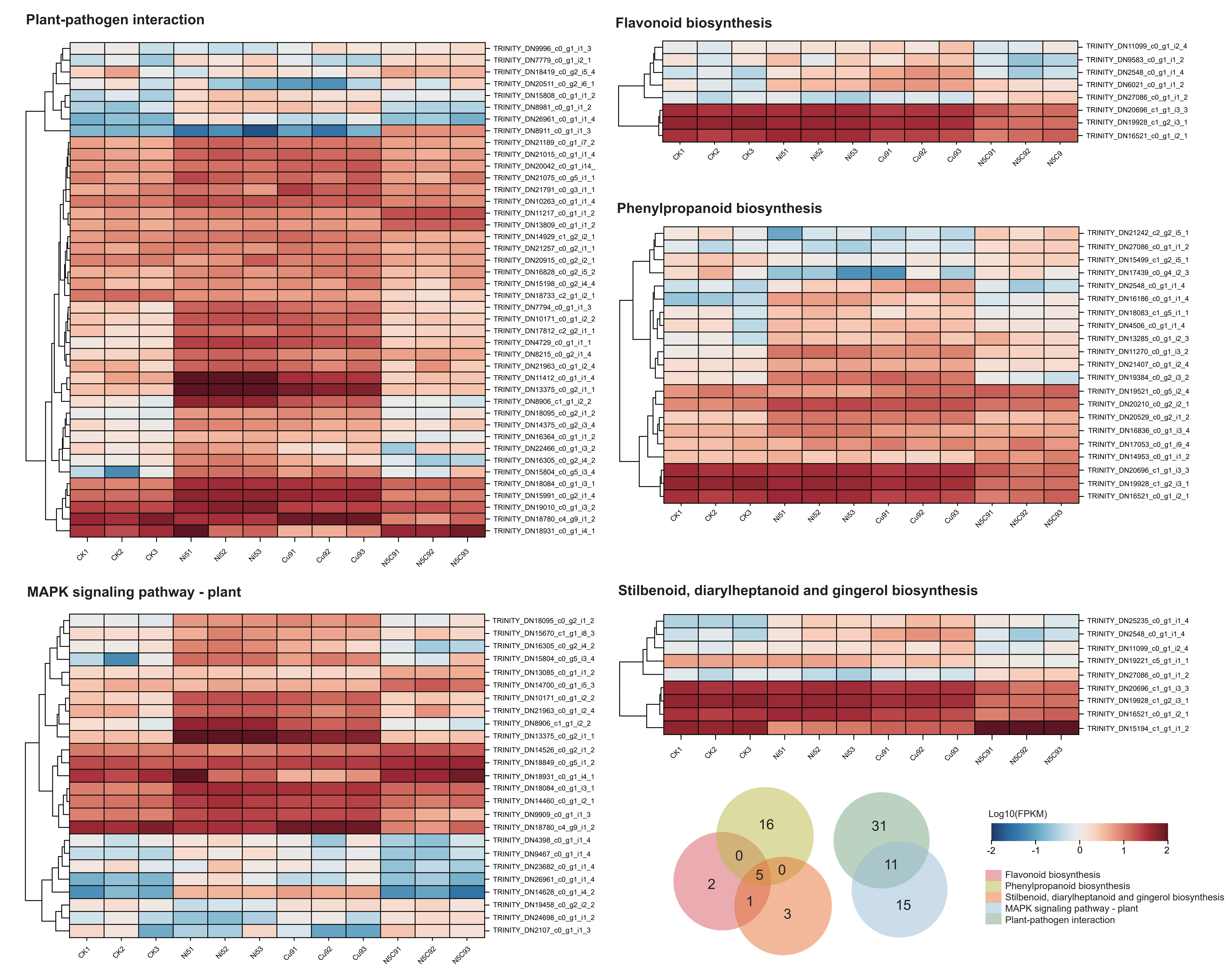
Plant-pathogen interaction
TRINITY_DN9996_c0_g1_i1_3
TRINITY_DN7779_c0_g1_i2_1
TRINITY_DN18419_c0_g2_i5_4
TRINITY_DN20511_c0_g2_i6_1
TRINITY_DN15808_c0_g1_i1_2
TRINITY_DN8981_c0_g1_i1_2
TRINITY_DN26961_c0_g1_i1_4
TRINITY_DN8911_c0_g1_i1_3
TRINITY_DN21189_c0_g1_i7_2
TRINITY_DN21015_c0_g1_i1_4
TRINITY_DN20042_c0_g1_i14_
TRINITY_DN21075_c0_g5_i1_1
TRINITY_DN21791_c0_g3_i1_1
TRINITY_DN10263_c0_g1_i1_4
TRINITY_DN11217_c0_g1_i1_2
TRINITY_DN13809_c0_g1_i1_2
TRINITY_DN14929_c1_g2_i2_1
TRINITY_DN21257_c0_g2_i1_1
TRINITY_DN20915_c0_g2_i2_1
TRINITY_DN16828_c0_g2_i5_2
TRINITY_DN15198_c0_g2_i4_4
TRINITY_DN18733_c2_g1_i2_1
TRINITY_DN7794_c0_g1_i1_3
TRINITY_DN10171_c0_g1_i2_2
TRINITY_DN17812_c2_g2_i1_1
TRINITY_DN4729_c0_g1_i1_1
TRINITY_DN8215_c0_g2_i1_4
TRINITY_DN21963_c0_g1_i2_4
TRINITY_DN11412_c0_g1_i1_4
TRINITY_DN13375_c0_g2_i1_1
TRINITY_DN8906_c1_g1_i2_2
TRINITY_DN18095_c0_g2_i1_2
TRINITY_DN14375_c0_g2_i3_4
TRINITY_DN16364_c0_g1_i1_2
TRINITY_DN22466_c0_g1_i3_2
TRINITY_DN16305_c0_g2_i4_2
TRINITY_DN15804_c0_g5_i3_4
TRINITY_DN18084_c0_g1_i3_1
TRINITY_DN15991_c0_g2_i1_4
TRINITY_DN19010_c0_g1_i3_2
TRINITY_DN18780_c4_g9_i1_2
TRINITY_DN18931_c0_g1_i4_1
CK1 CK2 CK3 Ni51 Ni52 Ni53 Cu91 Cu92 Cu93 N5C91 N5C92 N5C93
Flavonoid biosynthesis
TRINITY_DN11099_c0_g1_i2_4
TRINITY_DN9583_c0_g1_i1_2
TRINITY_DN2548_c0_g1_i1_4
TRINITY_DN6021_c0_g1_i1_2
TRINITY_DN27086_c0_g1_i1_2
TRINITY_DN20696_c1_g1_i3_3
TRINITY_DN19928_c1_g2_i3_1
TRINITY_DN16521_c0_g1_i2_1
CK1 CK2 CK3 Ni51 Ni52 Ni53 Cu91 Cu92 Cu93 N5C91 N5C92 N5C9
Phenylpropanoid biosynthesis
TRINITY_DN21242_c2_g2_i5_1
TRINITY_DN27086_c0_g1_i1_2
TRINITY_DN15499_c1_g2_i5_1
TRINITY_DN17439_c0_g4_i2_3
TRINITY_DN2548_c0_g1_i1_4
TRINITY_DN16186_c0_g1_i1_4
TRINITY_DN18083_c1_g5_i1_1
TRINITY_DN4506_c0_g1_i1_4
TRINITY_DN13285_c0_g1_i2_3
TRINITY_DN11270_c0_g1_i3_2
TRINITY_DN21407_c0_g1_i2_4
TRINITY_DN19384_c0_g2_i3_2
TRINITY_DN19521_c0_g5_i2_4
TRINITY_DN20210_c0_g2_i2_1
TRINITY_DN20529_c0_g2_i1_2
TRINITY_DN16836_c0_g1_i3_4
TRINITY_DN17053_c0_g1_i9_4
TRINITY_DN14953_c0_g1_i1_2
TRINITY_DN20696_c1_g1_i3_3
TRINITY_DN19928_c1_g2_i3_1
TRINITY_DN16521_c0_g1_i2_1
CK1 CK2 CK3 Ni51 Ni52 Ni53 Cu91 Cu92 Cu93 N5C91 N5C92 N5C93
MAPK signaling pathway - plant
TRINITY_DN18095_c0_g2_i1_2
TRINITY_DN15670_c1_g1_i8_3
TRINITY_DN16305_c0_g2_i4_2
TRINITY_DN15804_c0_g5_i3_4
TRINITY_DN13085_c0_g1_i1_2
TRINITY_DN14700_c0_g1_i5_3
TRINITY_DN10171_c0_g1_i2_2
TRINITY_DN21963_c0_g1_i2_4
TRINITY_DN8906_c1_g1_i2_2
TRINITY_DN13375_c0_g2_i1_1
TRINITY_DN14526_c0_g2_i1_2
TRINITY_DN18849_c0_g5_i1_2
TRINITY_DN18931_c0_g1_i4_1
TRINITY_DN18084_c0_g1_i3_1
TRINITY_DN14460_c0_g1_i2_1
TRINITY_DN9909_c0_g1_i1_3
TRINITY_DN18780_c4_g9_i1_2
TRINITY_DN4398_c0_g1_i1_4
TRINITY_DN9467_c0_g1_i1_4
TRINITY_DN23682_c0_g1_i1_4
TRINITY_DN26961_c0_g1_i1_4
TRINITY_DN14628_c0_g1_i4_2
TRINITY_DN19458_c0_g2_i2_2
TRINITY_DN24698_c0_g1_i1_2
TRINITY_DN2107_c0_g1_i1_3
CK1 CK2 CK3 Ni51 Ni52 Ni53 Cu91 Cu92 Cu93 N5C91 N5C92 N5C93
Stilbenoid, diarylheptanoid and gingerol biosynthesis
TRINITY_DN25235_c0_g1_i1_4
TRINITY_DN2548_c0_g1_i1_4
TRINITY_DN11099_c0_g1_i2_4
TRINITY_DN19221_c5_g1_i1_1
TRINITY_DN27086_c0_g1_i1_2
TRINITY_DN20696_c1_g1_i3_3
TRINITY_DN19928_c1_g2_i3_1
TRINITY_DN16521_c0_g1_i2_1
TRINITY_DN15194_c1_g1_i1_2
CK1 CK2 CK3 Ni51 Ni52 Ni53 Cu91 Cu92 Cu93 N5C91 N5C92 N5C93
16
0
2
5
0
1
3
31
11
15
Log10(FPKM)
-2 -1 0 1 2
Flavonoid biosynthesis
Phenylpropanoid biosynthesis
Stilbenoid, diarylheptanoid and gingerol biosynthesis
MAPK signaling pathway - plant
Plant-pathogen interaction
