# Supplementary figures and images for: Euphorbia marginata Alleviate Heavy Metal Ni-Cu Combined Stress by Regulating the Synthesis of Signaling Factors and Flavonoid Organisms
Source: Plants (Basel). 2025 Jul 13;14(14):2159. doi: 10.3390/plants14142159 (PMC12298763; doi:10.3390/plants14142159)

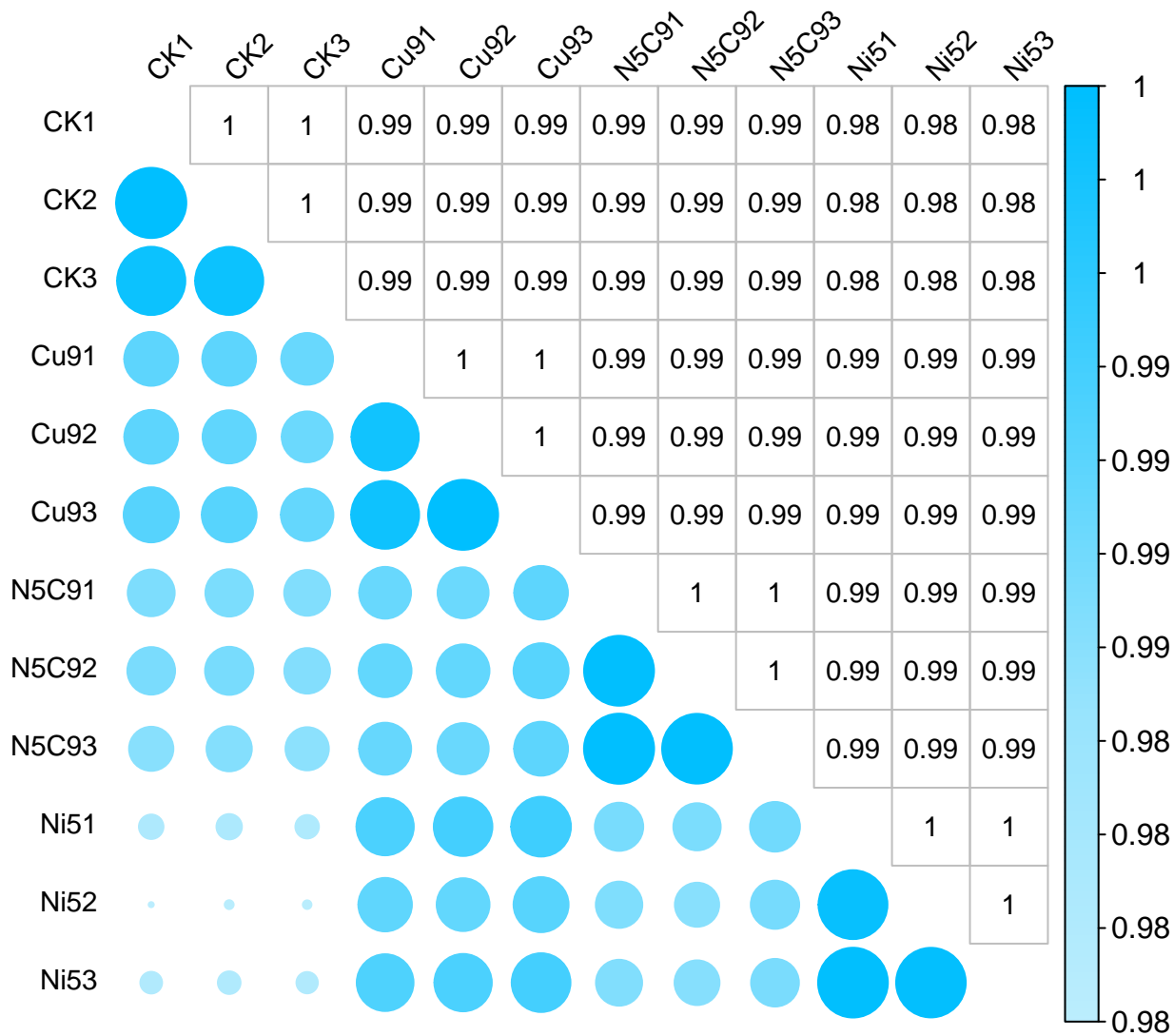

Supplement: Supplementary file 1 [file plants-14-02159-s001.zip › Figure S1. Correlation analysis between different samples.pdf]

# A4-vs-A2: q-value<0.05 && |log<sub>2</sub> FC|>1

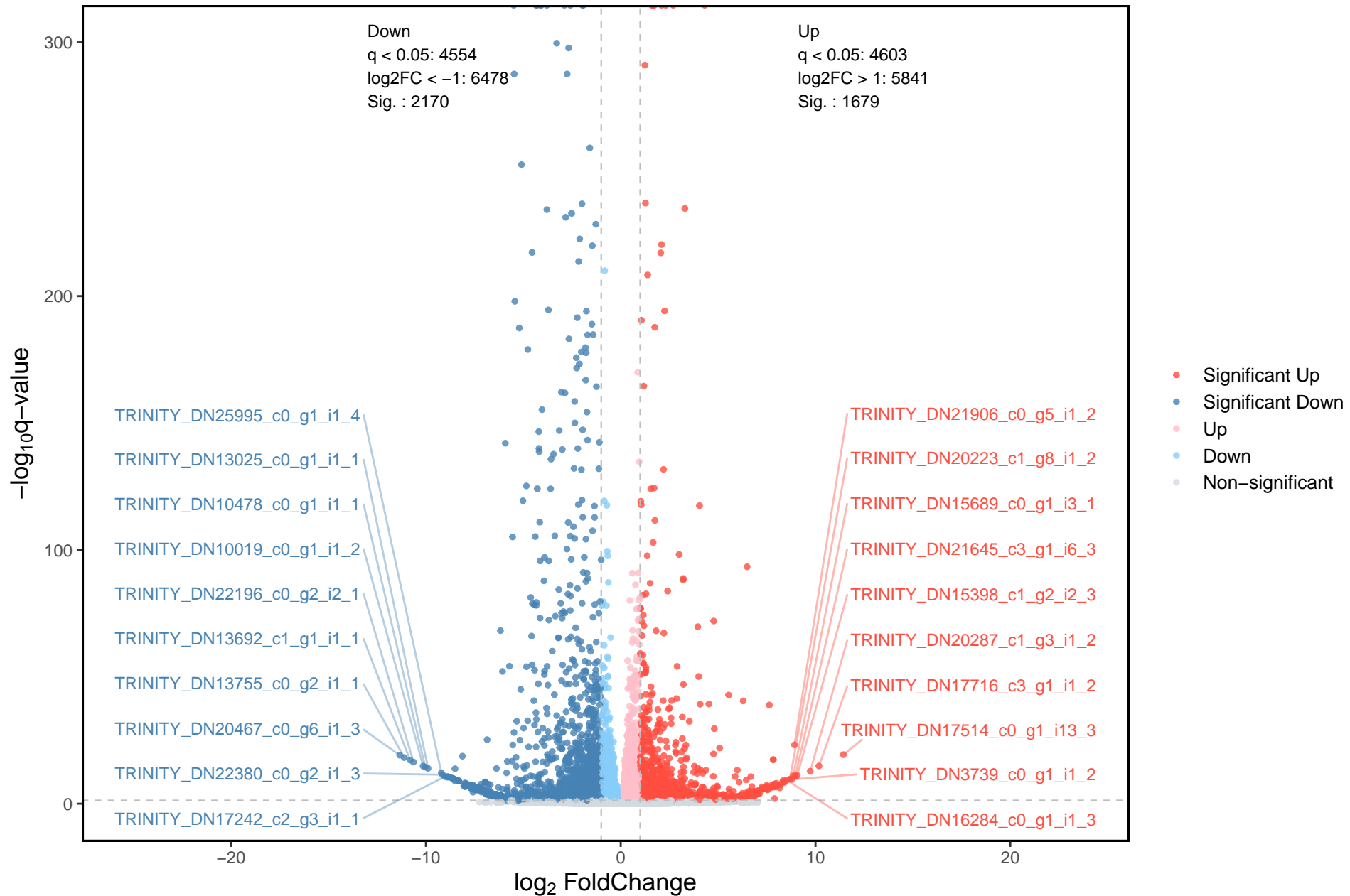

Supplement: Supplementary file 1 [file plants-14-02159-s001.zip › Figure S2. Volcano map of differential genes under Ni and combined stresses.pdf]

# A4-vs-A3: q-value<0.05 && |log<sub>2</sub> FC|>1

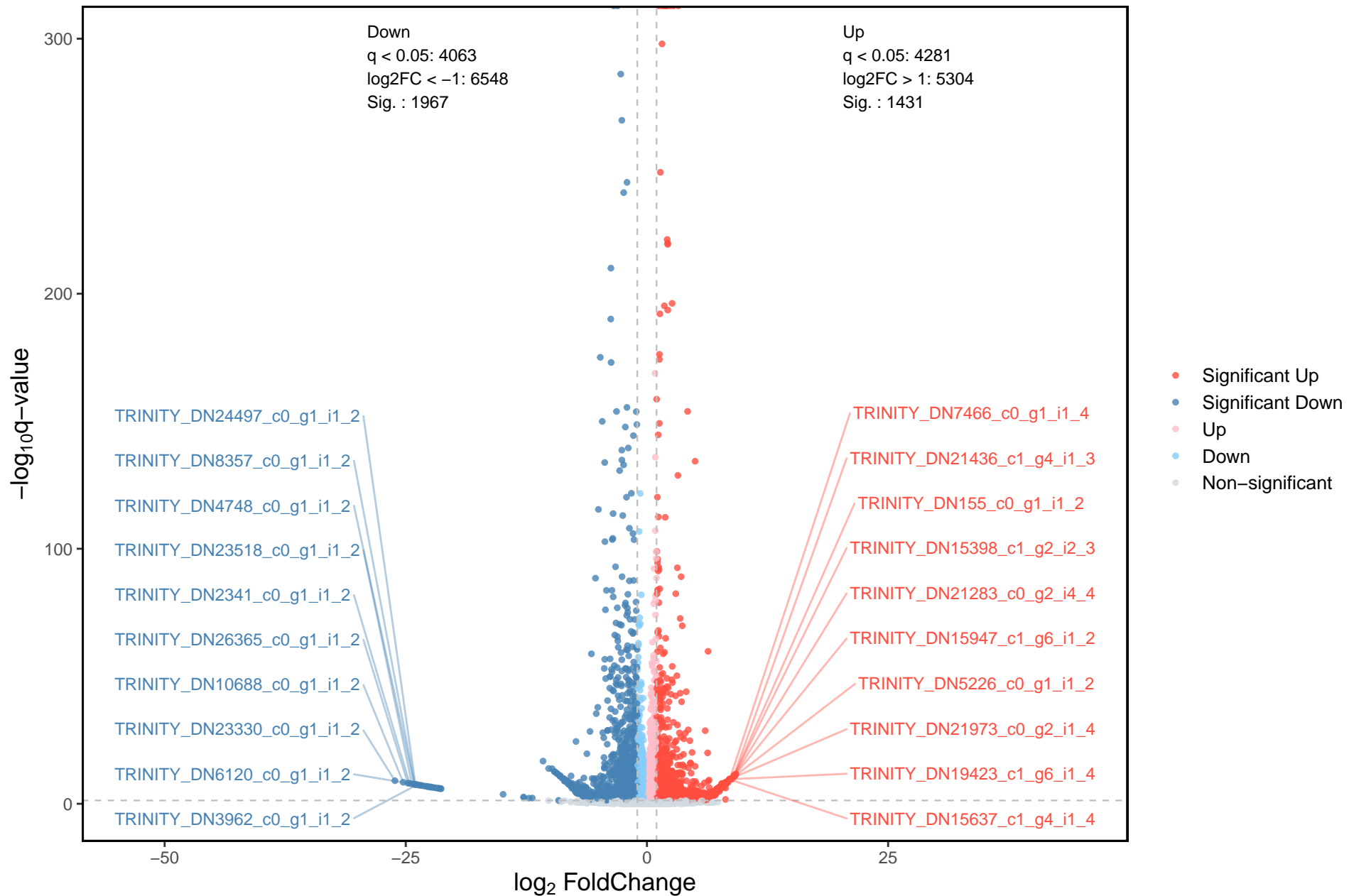

Supplement: Supplementary file 1 [file plants-14-02159-s001.zip › Figure S3. Volcano map of differential genes under Cu and combined stresses.pdf]

TRINITY\_DN18610\_c3\_g1\_i5\_3

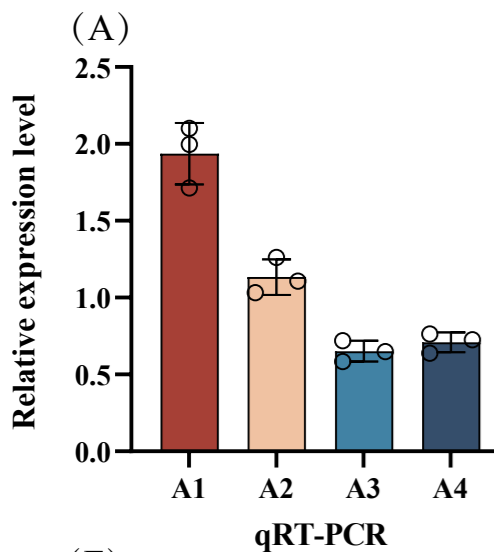

TRINITY\_DN14591\_c0\_g2\_i1\_3

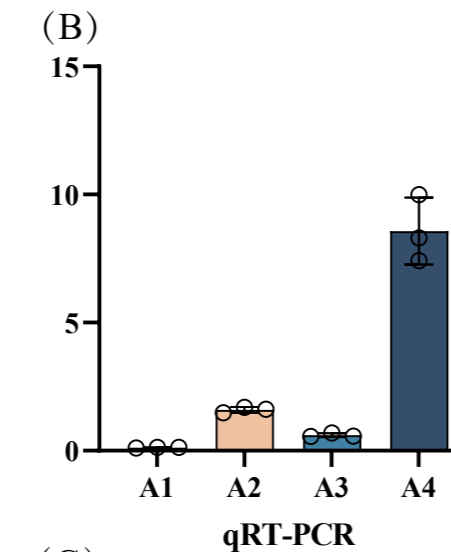

TRINITY\_DN13375\_c0\_g2\_i1\_1

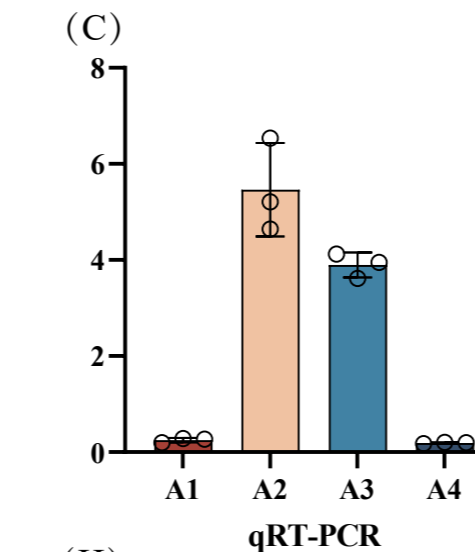

TRINITY\_DN11884\_c0\_g1\_i1\_1

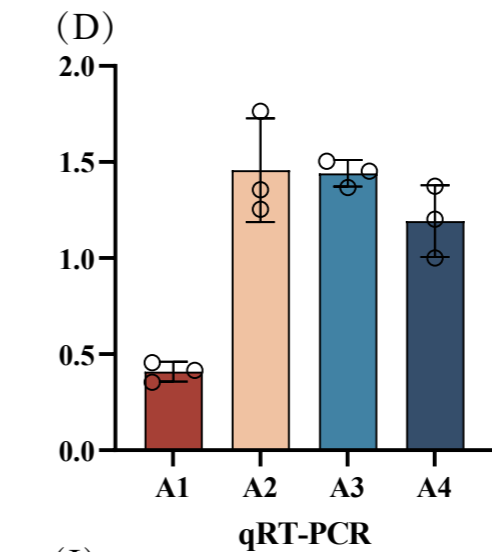

TRINITY\_DN11412\_c0\_g1\_i1\_1

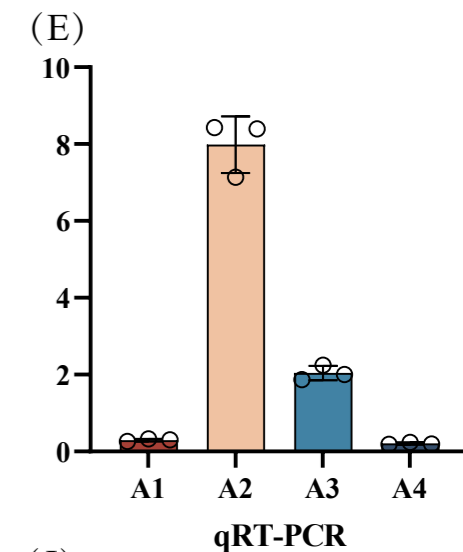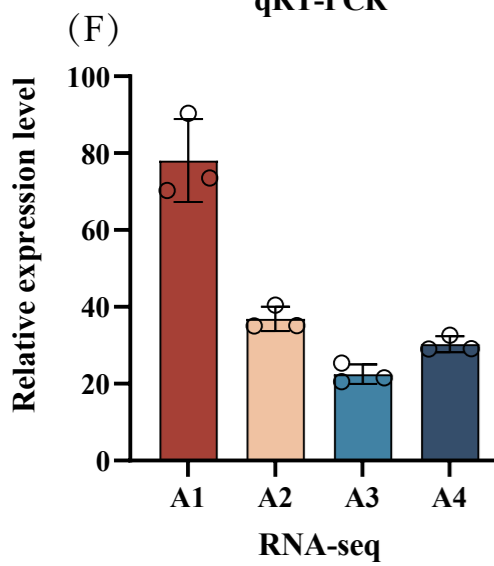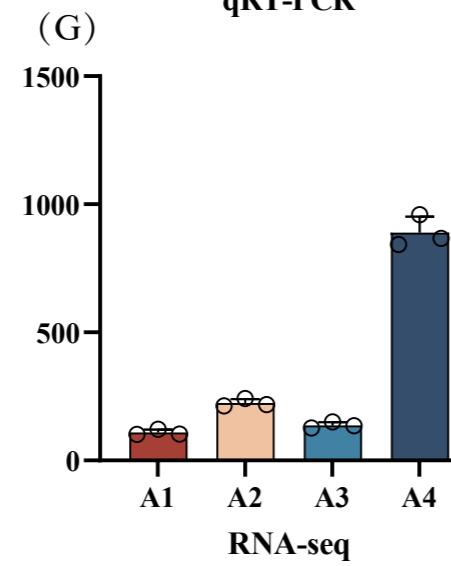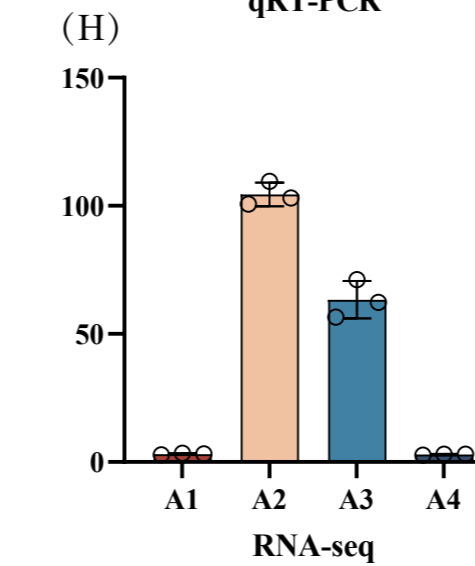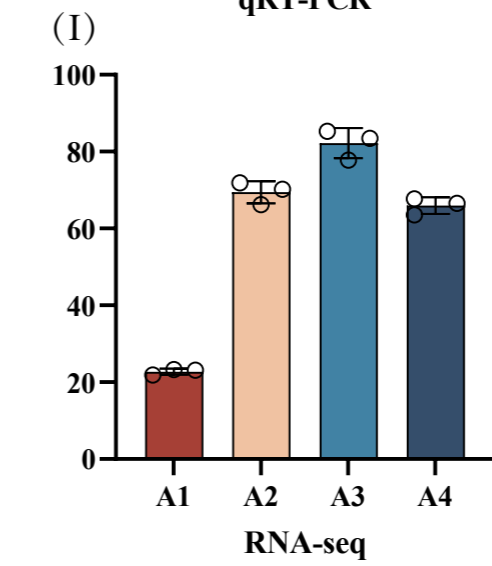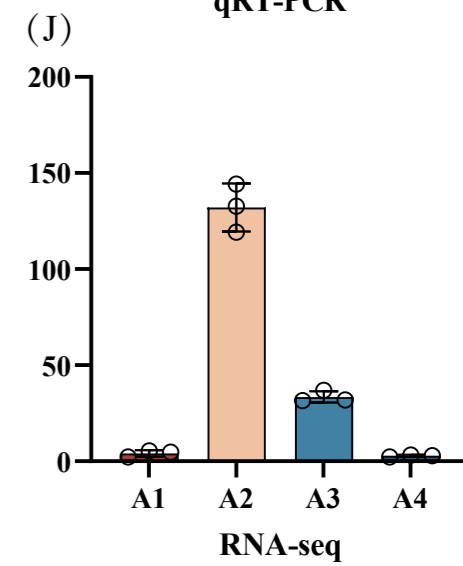

Supplement: Supplementary file 1 [file plants-14-02159-s001.zip › Figure S5. Validation of RNA-seq data by qRT-PCR showing concordant expression trends of 5 differentially expressed genes.pdf]
